# Automated processing of webcam images for phenological classification

**DOI:** 10.1371/journal.pone.0171918

**Published:** 2017-02-24

**Authors:** Ludwig Bothmann, Annette Menzel, Bjoern H. Menze, Christian Schunk, Göran Kauermann

**Affiliations:** 1 Institut für Statistik, Ludwig-Maximilians-Universität München, Munich, Germany; 2 Ökoklimatologie, Wissenschaftszentrum Weihenstephan für Ernährung, Landnutzung und Umwelt, Technische Universität München, Freising, Germany; 3 Institute for Advanced Study, Technische Universität München, Garching, Germany; 4 Department of Informatics, Technische Universität München, Munich, Germany; Pacific Northwest National Laboratory, UNITED STATES

## Abstract

Along with the global climate change, there is an increasing interest for its effect on phenological patterns such as start and end of the growing season. Scientific digital webcams are used for this purpose taking every day one or more images from the same natural motive showing for example trees or grassland sites. To derive phenological patterns from the webcam images, regions of interest are manually defined on these images by an expert and subsequently a time series of percentage greenness is derived and analyzed with respect to structural changes. While this standard approach leads to satisfying results and allows to determine dates of phenological change points, it is associated with a considerable amount of manual work and is therefore constrained to a limited number of webcams only. In particular, this forbids to apply the phenological analysis to a large network of publicly accessible webcams in order to capture spatial phenological variation. In order to be able to scale up the analysis to several hundreds or thousands of webcams, we propose and evaluate two automated alternatives for the definition of regions of interest, allowing for efficient analyses of webcam images. A semi-supervised approach selects pixels based on the correlation of the pixels’ time series of percentage greenness with a few prototype pixels. An unsupervised approach clusters pixels based on scores of a singular value decomposition. We show for a scientific webcam that the resulting regions of interest are at least as informative as those chosen by an expert with the advantage that no manual action is required. Additionally, we show that the methods can even be applied to publicly available webcams accessed via the internet yielding interesting partitions of the analyzed images. Finally, we show that the methods are suitable for the intended big data applications by analyzing 13988 webcams from the AMOS database. All developed methods are implemented in the statistical software package R and publicly available in the R package phenofun. Executable example code is provided as supplementary material.

## Introduction

The last years have seen an increasing interest in using webcam images to monitor and derive phenological patterns and their variation in time and space. Phenology, which describes the timing of annually recurring events in nature, has been recognized to be a key factor in ecosystem processes. The dates of the start and end of the growing season define the length of the vegetation period, then triggering biogeochemical fluxes between atmosphere and biosphere, such as carbon sequestration [1, 2], ecosystem respiration [1, 3], and biomass production [4]. Moreover, the seasonality of vegetation activity determines vegetation cover and biodiversity, but also controls the vegetation feedbacks to the climate system, in terms of oxygen production, evapotranspiration, BVOC emission, and other surface layer changes [2, 5–7]. Phenological information is either collected by volunteers or semi-professionals in phenological networks run by national meteorological services or citizen science organizations [8]. At this local scale that is also covered by ground based webcams, onset dates of leaf unfolding or leaf coloring are recorded for selected sites and species.

At the regional and even national and continental scale, start and end of season dates can also be derived from annual time series of vegetation indices from various remote sensing products, mainly AVHRR, MODIS, and MERIS instruments. The longest available record is the Normalized Difference Vegetation Index (NDVI) with data from 1982 onwards [9, 10]. However, remote sensing only provides a coarser spatial and temporal resolution, the so-called integrated land surface phenology (LSP) [11]. For example, using the so-called maximum-value composite procedure [12] with a MODIS instrument, the resulting resolution is 250*m* × 250*m* per pixel and one observation every 16 days. Unfortunately, linking LSP with species- and site-specific ground observation has turned out to be quite complicated or even impossible [13–15].

To close the gap between LSP and species- and site-specific ground observations, close surface remote sensing in terms of daily digital camera images has been proposed [16], see also [17–20]. A major intention is to automatically capture seasonal changes on a fine spatial resolution, which could be at the end also on species-level. Secondly, one wants to relate the findings with satellite remote sensing systems, since the percentage of greenness (%greenness) derived from the RGB information mirrors the temporal behavior of vegetation indices by remote sensing, see e.g. [21]. Still, single scientific cameras will not yield spatially dense information. Therefore, a dense webcam network is required which then demands for advanced and automatic image processing in order to handle these high resolution spatio-temporal data.

Usually, webcams are used for the identification of phenological patterns as follows: A webcam takes every day one or more images from the same natural motive showing for example trees or grassland sites [20, 22]. Based on these images, the dates of (1) the start of the growing season (SOS), (2) the point of maximum %greenness (MAX) and (3) the end of the growing season with start (EOS1) and end (EOS2) of leaf coloring in autumn shall be determined. Therefore, the dimension of the data is reduced by first defining regions of interest (ROIs) on the image and then computing a %greenness time series in these regions. Finally, the dates of SOS, MAX, EOS1 and EOS2 are determined by a search for structural changes in the %greenness time series, see for example [19, 23, 24].

While this procedure is suitable for determining the dates of SOS, MAX, EOS1 and EOS2, it is associated with a considerable amount of manual work, because the ROIs have to be defined manually by an expert. We call these regions “expert-based regions of interest” (**eROI**) in the remainder to differentiate this standard approach from our new approaches for the definition of ROIs. Our aim is to use webcam images on a larger scale and to analyze the data of several hundreds or thousands of webcams in short time. For this purpose, the processing of the webcam images has to be automated and an efficient implementation is needed. Instead of defining ROIs manually, we propose two data-driven approaches:

- First, we propose a **semi-supervised** approach: We select a very small number of pixels which clearly show phenological features like deciduous trees. This is in principle done as in the eROI approach, but here we just select a few number of pixels (6 × 6) rather than delineating the structures of interest at fine resolution as done in the eROI approach. Once these pixels of interest are defined, we let them grow to a data-driven region of interest by adding pixels with high seasonal correlation. We call this region “semi-supervised region of interest” (**sROI**) in the remainder.
  This strategy is an improvement of the standard approach because instead of defining large expert-based eROIs we just set a pinprick on the image which requires less manual effort from the expert and may even be applied by non-experts.
  Additionally, we provide a fully automated and purely data-driven version of the sROI approach: Instead of defining a single expert-chosen pinprick, multiple random pinpricks can be placed on the image. Then, based on two optimality criteria defined later in this paper, the best sROI in terms of phenological information can be selected.
- As second strategy we propose an **unsupervised** approach: We carry out a singular value decomposition (SVD) of the images in order to reduce the dimension of the data while maintaining the information about phenological variation. A subsequent cluster analysis groups the pixels with regard to their variation over time. Finally, we identify the interesting clusters of pixels which show the highest phenological variation by comparing the above mentioned optimality criteria for the resulting %greenness time series. The resulting cluster of pixels is called “unsupervised region of interest” (**uROI**) in the remainder.

Both approaches lead to ROIs for the images. These ROIs can be used to derive and analyze %greenness time series as commonly done in the phenological community. This means, structural changes in these time series can be searched for and thus dates of SOS, MAX, EOS1 and EOS2 are identified. We show that with our more advanced definitions of ROIs we obtain %greenness time series which carry at least as much phenological information as those based on the standard eROI approach. The advantage of our approach is that considerably less manual effort is required. By analyzing 13988 webcams of the AMOS database (http://amos.cse.wustl.edu/) we show that the methods allow for big data applications.

## Data and methods

In this section, we first give a short overview of the structure of the data we are working with. Then we propose two alternatives to the standard approach of defining expert-based regions of interest (eROI), namely the “semi-supervised regions of interest” (sROI) and the “unsupervised regions of interest” (uROI).

### Data description

We demonstrate our routines with a scientific webcam and three publicly available webcams. Additionally, we show that the methods can be used on a larger scale as well by applying them to a database of several thousands of webcams.

**Fig 1:**
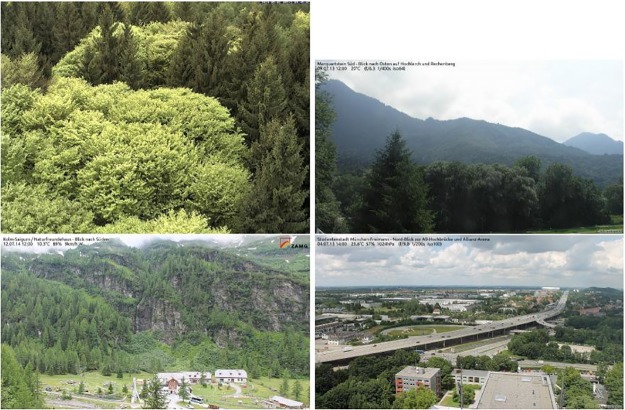
Example webcam images. Images of Kranzberg forest on DOY 121 (top left), Marquartstein on DOY 190 (top right), Kolm-Saigurn Sonnblickbasis on DOY 193 (bottom left) and Studentenstadt München-Freimann on DOY 185 (bottom right).

During the year 2014, a scientific MOBOTIX M12D-Sec-DNight camera recorded four images per day with the same field of view. The study site Kranzberg Forest (mixed Fagus sylvatica L./Picea abies [L.] Karst. forest) is located near Freising, Germany at 48°25’08” N and 11°39’41” E (485 m.a.s.l.). Two images were taken in the morning around 9.45 am and two images were taken on midday around 1.45 pm. Due to technical reasons, some images had to be deleted, resulting in a total of 1441 images. The image resolution is 1276 × 960 pixels, Fig 1 (top left) shows an example image from DOY (day of year) 121. The methods in this section will be explained based on these data.

The first open-access webcam data were taken from the website http://www.foto-webcam.eu. Here, we use three sites: First, data from Marquartstein Süd, near the lake Chiemsee in South Germany at 47°44’60” N and 12°27’36” E; second, data from Kolm-Saigurn, Austria at 47°04’10” N and 12°59’04” E; and third, data from the Studentenstadt in the north of Munich, Germany at 48°11’02” N and 11°36’41” E. Example images are shown in Fig 1. The webcams were selected to cover a wide range of possible views, i.e. from rich phenological information (Marquartstein and Kolm-Saigurn, top right and bottom left) to poor phenological information looking at buildings and streets in a big city (Studentenstadt, bottom right). All images have a resolution of 1200 × 675 pixels where the top 50 pixel rows of each image were discarded before the analysis to delete the time stamp and location information. At each site we use one image per day taken around midday.

**Fig 2:**
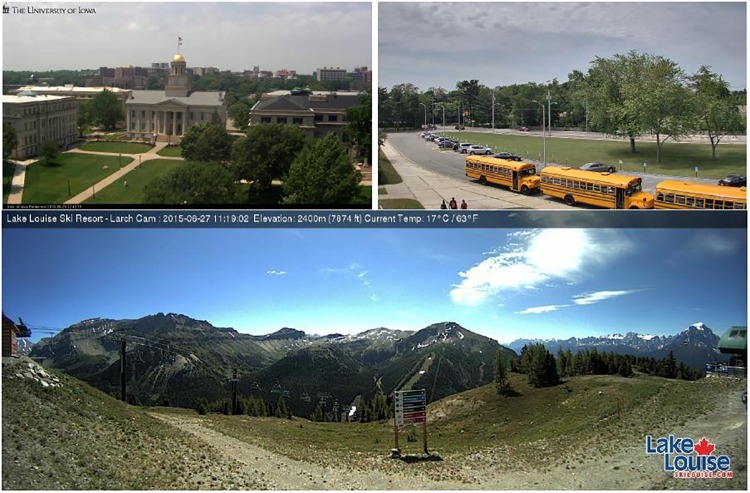
Example webcam images for three selected webcams from AMOS. Images are available at http://amos.cse.wustl.edu/.

AMOS—the archive of many outdoor scenes “is a collection of long-term timelapse imagery from publicly accessible outdoor webcams around the world” [25] and can be accessed through http://amos.cse.wustl.edu/. It contains image data from almost 30000 webcams of which the majority is located in the United States. Among others, it collects data from the PhenoCam network, see https://phenocam.sr.unh.edu/webcam/. A map with the locations of all cameras can be found at http://amos.cse.wustl.edu/browse_map. Fig 2 shows example images for camera ID 8221 from Iowa, USA at 41°39’41” N and 91°32’06” W (top left image), camera ID 441 from New York, USA at 40°46’50” N and 73°11’49” W (top right image) and camera ID 22231 from Lake Louise, Alberta, Canada at 51°26’37” N and 116°09’30” W (bottom image). The first two cameras show deciduous trees and grassland in large parts of the images. Here, the challenge for the algorithm is not to mix up pixels of deciduous trees and grassland. The third camera shows no deciduous trees at all. Here, seasonality has to be derived from the grassland only.

### Expert-based ROI approach (eROI)

**Fig 3:**
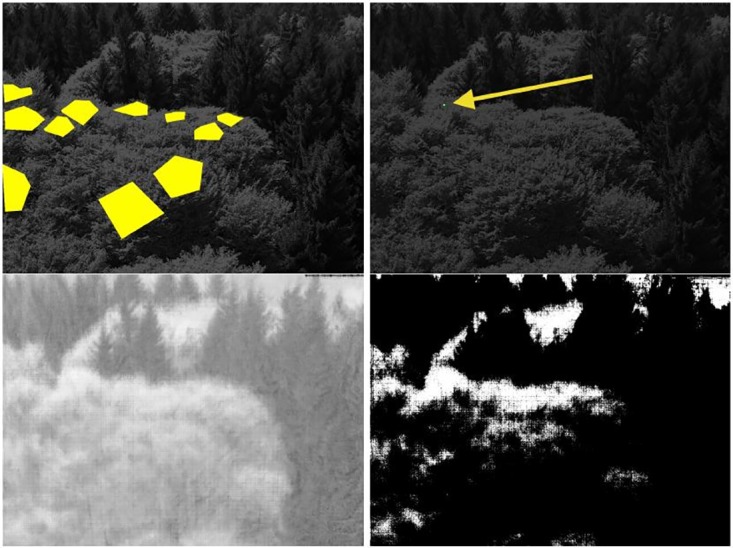
Expert-based eROI (top left), pinprick (top right), correlation image (bottom left, where brighter colors indicate higher correlations) and resulting sROI (bottom right) for Kranzberg data.

The standard approach for filtering the relevant information from the data is the following. The analyzing researcher defines one or more ROIs per hand by simply drawing rectangles, polygons or free shapes around regions which are subjectively relevant, usually around crowns or canopies of deciduous trees. Deciduous trees are of special interest because they show a high seasonal variation necessary for phenological classification, whereas evergreen trees display less; other abiotic items, such as bare soil, stone and sky should not be affected. This means, a ROI is generated that defines which pixels are discarded and which pixels remain in the data for further analyses. Fig 3 (top left) shows the “expert-based regions of interest” (eROI) for the scientific webcam as example. These eROIs comprise parts of the upper sun crown, however, specifically for individual specimens, thus their sizes are relatively small. These eROIs in the individual tree crowns were identified as polygonal areas with the help of two persons, one at the ground checking the crown-individuum correspondence and one in a crane gondola, maneuvered upon the specific parts to mark them for further identification in the digital images.

Such an approach is state-of-the-art and yields satisfying results, since it allows for the identification of single trees even in an homogeneous stand of one species, see for example [19, 20, 24]. Nevertheless, this approach requires an expert to define the eROIs manually. Therefore, it does not allow to be scaled up to an analysis of hundreds or thousands of webcams where the focus would be on the phenology of general vegetation types (e.g. deciduous trees, evergreen vegetation).

### Semi-supervised ROI approach (sROI)

The choice of eROIs can be subjective and the results may be improved by a more sophisticated and data-driven approach which we call “semi-supervised regions of interest” (sROI). The term semi-supervised refers to the fact that we start with a few handpicked pixels. Then, the region of interest grows data-driven to capture most of the relevant information in the images. The sROI approach works as follows.

**Fig 4:**
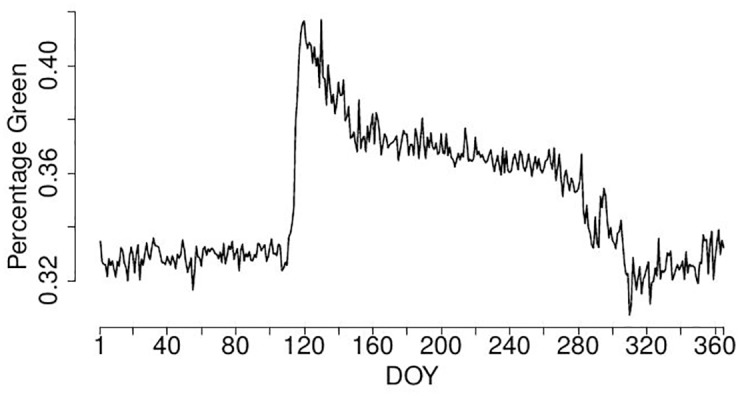
%greenness in pinprick over the year, resulting time series from Kranzberg data.

First, we select a very small ROI in the middle of the crown of a deciduous tree, for example 6 × 6 pixels. This means that we point with a needle on the picture, where the needle head is on a clearly identifiable deciduous tree. This pinprick is visualized in Fig 3 (top right). Second, for the selected few pinprick pixels, we compute the %greenness time series, shown in Fig 4. As third step we compute for each pixel in the image the %greenness time series over the year and correlate it with the pinprick pixels’ %greenness time series using pearson’s correlation coefficient. This results in a correlation image as shown in Fig 3 (bottom left). The brighter the level of gray is, the higher is the pixel’s correlation with the pinprick. As final step, we threshold this correlation image and discard all pixels which have small correlations, in the example correlations of less than 0.65 are neglected, see Fig 3 (bottom right).

In the example given above, the pinprick and the correlation threshold were chosen manually to obtain a visually optimal ROI. Since we are heading towards an automated approach, we propose the following extension: Instead of setting a single pinprick expert-based in the crown of a deciduous tree, we distribute *q* pinpricks randomly over the image. Additionally, the threshold for the correlation image is not chosen subjectively as above but we compute candidate ROIs for a grid of *m* correlation thresholds between 0 and 1. This leads to a number of *qm* candidate ROIs, i.e. one for each combination of pinprick and correlation threshold. Now, the crucial question is: How can we select the best ROI from these *qm* candidate ROIs? We propose two optimality criteria which can be used for this decision.

#### Optimality criterion 1

The first optimality criterion (OC1) is based on methods from the field of structural change point analysis. Since we are searching for the pixels with the highest phenological variation over time, we know that the corresponding %greenness time series has to show a significant structural change in spring. Before and after this change, the %greenness time series has to be more or less linear until autumn. We use this knowledge to define an optimality criterion as follows.

We only consider the first 240 days and thereby delete the autumn and beginning of winter. First, we compute the %greenness time series of the pixels inside the ROI. Then we search for a structural change in this %greenness time series with usual methods for structural change point detection, for example described in [26–28] and implemented in the R package strucchange by [29]. All time points from DOY 30 to DOY 210 are subsequently considered as potential change point. For a given change point, a linear model is fitted with changing intercept and slope at the change point. Additionally, a linear model without change point is fitted and then, an F-statistic is computed to compare the model with the change point with the model without the change point:

$$\begin{array}{c} F=\frac{(RSS_{0}-RSS_{1})/k}{RSS_{1}/\left(n-2k\right)}, \end{array}$$

where *RSS*_0_ and *RSS*_1_ are the residual sum of squares for the model without change point and with change point, respectively, *k* is the number of estimated parameters in the model without change point, i.e. here *k* = 2, and *n* is the number of time points, i.e. here *n* = 240. See also [29] for details.

Finally, the change point with the largest F-statistic is considered as the point of structural change, and the value of the largest F-statistic is the value of the optimality criterion OC1 for this time series and corresponding ROI.

Since we are searching for a structural change in each of the *qm* %greenness time series, we obtain *qm* change points with *qm* values of the F-statistic. The best combination of pinprick and correlation threshold is that one leading to the largest F-statistic because for this threshold, the signal-to-noise ratio in the resulting %greenness time series is maximal with respect to the fitted models. The corresponding ROI is the final sROI.

#### Optimality criterion 2

The second optimality criterion (OC2) is closer to the expected seasonal pattern of the %greenness time series. From long-term phenological experience we know that the %grenness time series of a deciduous tree behaves as follows: In winter, the time series stays constant on a rather low level. In spring, there is a steep increase followed by a small decrease and then, in summer, the time series remains again constant but on a higher level than in winter. Finally, in autumn, the time series further decreases until the level of winter is reached.

**Fig 5:**
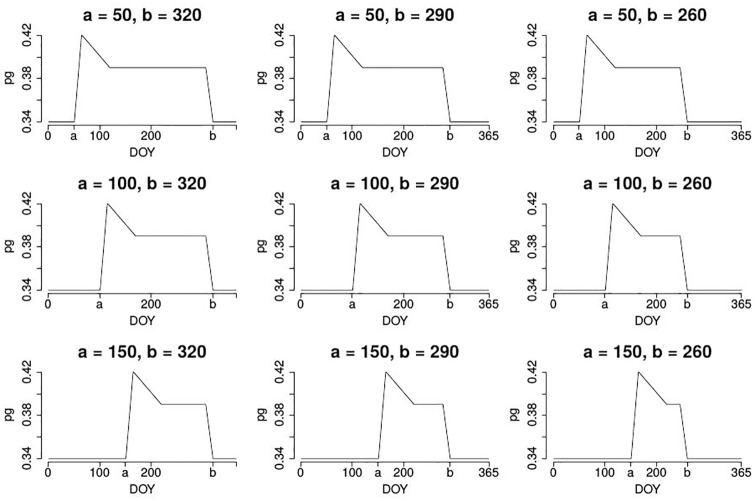
Template %greenness time series for varying values of *a* and *b*.

Although we know this rough pattern, we do not know the concrete dates of the change points. But we can exploit our structural knowledge: We define a large set of template time series which reflect the known seasonal patterns but differ in the time points of starting spring *a* and ending autumn *b*. Fig 5 shows nine of these template time series for varying values of *a* and *b*. In principle also other templates such as grassland systems, agricultural cereals or infrastructure could be used. Alternatively, the template time series for deciduous trees could be allowed to be more flexible by additionally varying parameters *c*, *d* and *e* for the points of maximum %greenness, begin and end of the summer plateau.

For a given ROI, we can now compute the correlation of the resulting %greenness time series with each of the template time series, where the values *a* and *b* vary in all possible combinations between *a* ∈ {50, 51, … 149, 150} and *b* ∈ {265, 266, … 364, 365}. Then, the highest correlation defines the value of the optimality criterion OC2 for the given ROI.

Since we are searching for the best combination of *q* pinpricks and *m* correlation thresholds, we obtain *qm* values for OC2. The best combination of pinprick and correlation threshold is that one leading to the largest OC2. The corresponding ROI is the final sROI. This approach has the advantage that additionally to the definition of an optimality criterion, we obtain rough estimates of the SOS and EOS2 dates via the values *a* and *b* for the best combination.

Thus, there are two options when using the sROI procedure: A semi-automated and an automated version. For the semi-automated version, a single prinprick has to be set by an expert. With this pinprick, the sROI procedure computes an optimal ROI by optimizing the correlation threshold. For the fully automated version, *q* pinpricks are randomly distributed over the image. Then, for each pinprick the best threshold is found and finally the best combination of pinprick and correlation threshold is identified. This defines the final sROI.

### Unsupervised ROI approach (uROI)

We propose an alternative approach for the automated definition of ROIs, called “unsupervised regions of interest” (uROI).

We assume the following data structure of the color images: We consider each color channel of each pixel as observation unit and each time point *t* = 1, …, *T* as variable. Each image has *n* = *n*_1_ ⋅ *n*_2_ = 1276 ⋅ 960 = 1.224.960 pixels, this leads to *T* = 1441 observations of 3*n* pixel color channels. We store each image in a vector of length 3*n* and denote it as ***x***_*t*_ = (*r*_1*t*_, …, *r*_*nt*_, *g*_1*t*_, …, *g*_*nt*_, *b*_1*t*_, …, *b*_*nt*_)^⊤^ for *t* = 1, …, *T* where *r*_*it*_, *g*_*it*_ and *b*_*it*_ denote the intensity of the red, green and blue color channel of pixel *i* at time *t*, respectively. Altogether we get a data matrix ***X*** = (***x***_1_, …, ***x***_*T*_) of dimension 3*n* × *T* which is centered prior to the following steps.

**Fig 6:**
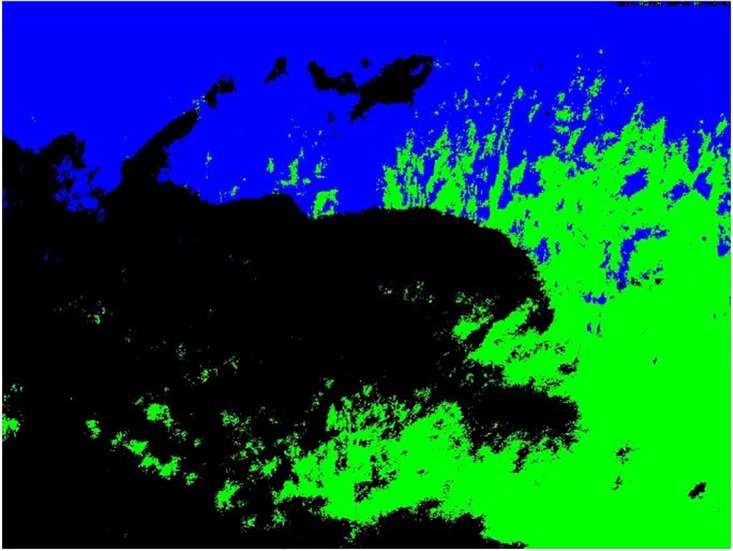
Resulting three clusters from uROI method. Cluster 1 in green, cluster 2 in blue and cluster 3 in black, to compare with Fig 1 (top left image). Kranzberg data 2014.

On ***X*** we perform a singular value decomposition (SVD). This means, ***X*** is decomposed into ***X*** = ***U*** ***D*** ***V***^⊤^, where the columns of ***U*** are the eigenvectors of ***X*** ***X***^⊤^, the columns of ***V*** are the eigenvectors of ***X***^⊤^ ***X*** and ***D*** contains the square roots of the non-zero eigenvalues of ***X*** ***X***^⊤^ or ***X***^⊤^ ***X***. In order to achieve a dimension reduction, we make use of a truncated version of the SVD and only compute the first *p* left singular vectors, i.e. the matrix ***U*** is of dimension 3*n* × *p*. The columns of ***U*** may also be called eigenimages. We rearrange rows and columns of ***U*** such that each pixel is described by 3*p* variables, i.e. *p* variables per color channel and denote it as U* with dimension *n* × 3*p*. Now that each pixel is described by a small set of only 3*p* variables, we can find similar pixels by performing standard cluster analyses on the rows of U*. While hierarchical clustering is computationally unfeasible due to the large number of pixels, k-means procedures lead to good results with low computational effort. As a result, the image is segmented into *k* clusters and each cluster of pixels defines a candidate ROI. Fig 6 shows the result of the cluster analysis for *p* = 12 and *k* = 3 for the Kranzberg data.

Comparing the resulting *k* = 3 clusters with the example image in Fig 1, we can see that cluster 3 (black) is candidate for the final ROI since it reflects the deciduous trees. Cluster 1 and 2 refer to evergreen Norway spruce specimens of which cluster 1 trees in the foreground clearly reveal spring seasonality due to May shoots as well as edge effects by the deciduous European beech trees.

**Fig 7:**
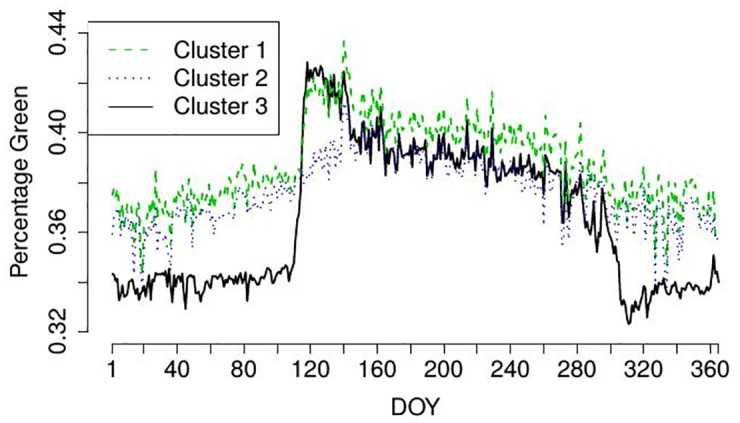
Resulting %greenness time series for clusters from uROI approach shown in Fig 6.

However, as we seek for a fully automated procedure, we select the final uROI data-driven and automatically. Therefore, we compute again the optimality criteria OC1 and OC2 for the %greenness time series for each cluster shown in Fig 7. In our example, the OC1 for the three clusters are 665, 299 and 1863, respectively and the OC2 for the three clusters are 0.89, 0.81 and 0.96, respectively. Thus, both optimality criteria clearly select cluster 3 as final uROI as expected by comparing the clusters with the original images.

The computational burden of the uROI approach is rather small, although an SVD of a very large matrix has to be carried out. Using the statistical software R [30], the singular vectors can be computed very efficiently with the package irlba [31].

In the results section we will investigate the performance of the approach and compare it with the results based on the eROI and sROI approaches. Phenological onset dates are derived from the %greenness time series of eROI, sROI and uROI. Change points in these time series are modelled using the multiple Bayesian change point approach as proposed by [19], based on [32, 33], and e.g. applied in [24, 34].

## Results

In this section we compare the eROI, sROI and uROI approaches for the definition of ROIs with the goal to identify the SOS, MAX, EOS1 and EOS2 dates. First, we use the images from the scientific camera at Kranzberg forest, second we use the images from the open-access webcam data from http://www.foto-webcam.eu and then we challenge the approach and analyze images from several thousands of webcams from AMOS (http://amos.cse.wustl.edu/).

### Scientific webcam

**Fig 8:**
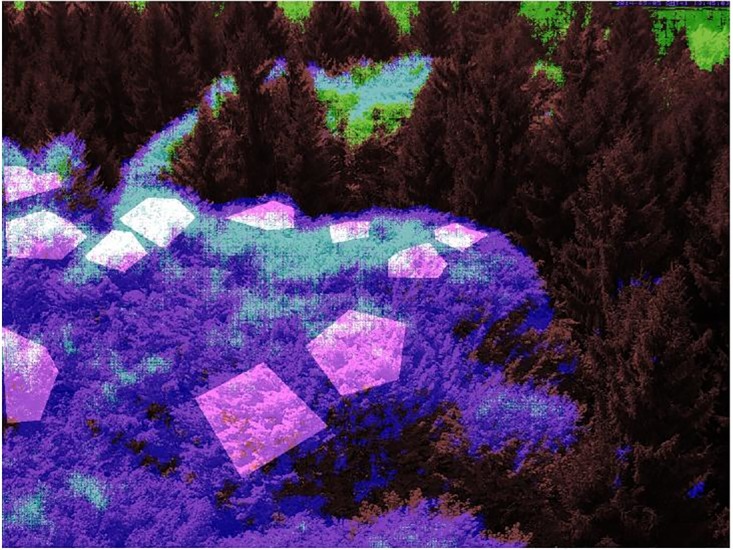
Overlay of the ROIs resulting for the eROI (pink polygons), sROI (green) and uROI (purple, overlap with sROI light blue) approaches.

Fig 8 shows an overlay of the ROIs resulting from the eROI, sROI and uROI approaches. For the sROI approach, one expert pinprick is used, both sROI and uROI use optimality criterion 2. All approaches exclude the evergreen trees but differ in the amount and selection of pixels corresponding to deciduous trees. In the following our aim is to decide which of these ROIs is optimal with respect to phenological information.

**Fig 9:**
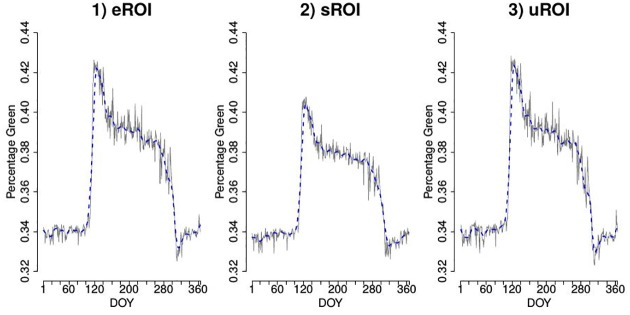
%greenness time series for three different approaches. Blue dashed lines show LOESS estimators for smoothed time series.

**Fig 10:**
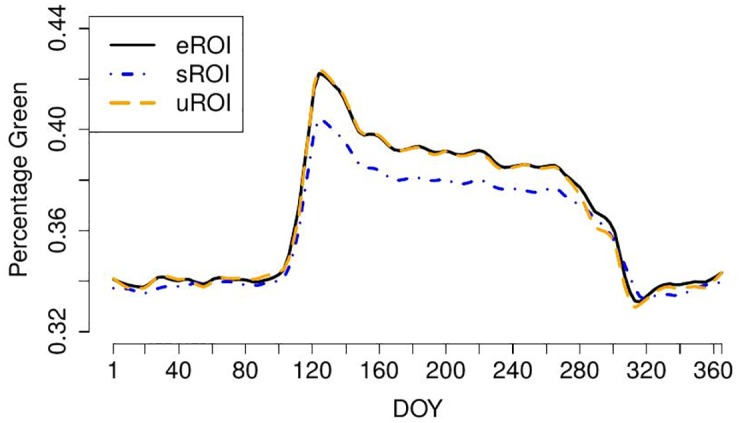
Comparison of LOESS estimators for smoothed %greenness time series for the images from Kranzberg forest.

The resulting %greenness time series for eROI, sROI and uROI for the data of Kranzberg 2014 are shown in Fig 9, see also Fig 10 for a comparison of the LOESS estimators. It seems that the time series resulting from the uROI and eROI approach are the most informatives because the decisive spring amplitude is slightly larger than for the sROI approach. However, the error variability is slightly smaller for the sROI approach. Thus, we need an objective measure to decide which time series is the most informative and therefore use the optimality criteria OC1 and OC2. The resulting values for OC1 and OC2 are shown in Table 1. It can be seen that the amount of information contained in the three time series is approximately the same, but the sROI time series carries the most information, followed by uROI and eROI.

**Table 1:** Values of optimality criteria OC1 and OC2 for different ROI approaches for images from Kranzberg data 2014.

	eROI	sROI	uROI
OC1	1848	1944	1863
OC2	0.967	0.972	0.961

For the identification of the SOS, MAX, EOS1 and EOS2 dates we use the approach of [19] and [24], see also [23, 35]. There, the dates of change points in the univariate %greenness time series are found via a Bayesian multiple change point analysis. The total number of change points is not specified by the researcher but determined automatically by the method. More traditional approaches are for example discussed in [36–39]

In all three cases eROI, sROI and uROI, four change points turned out to be optimal to describe the annual course of %greenness comprising SOS, MAX, EOS1 and EOS2. Table 2 shows the results.

**Table 2:** Estimated phenological change points (DOYs) for different ROI approaches for images from Kranzberg data 2014.

	eROI	sROI	uROI	Date
SOS	105.0	105.0	105.1	April 15
MAX	119.0	119.0	118.9	April 29
EOS1	303.0	303.0	302.8	October 30
EOS2	308.0	308.0	307.7	November 4

Thus, the information about phenological variation resulting from eROI, sROI and uROI are almost identical, both regarding the optimality criteria OC1 and OC2 and regarding the estimated phenological change points. Therefore, we conclude that the sROI and uROI approaches outperform the approach of expert-based eROIs because additionally to the degree of information contained in the %greenness time series, they have the great advantage that they are fully automated.

### Open-access webcams - foto-webcam.eu

**Fig 11:**
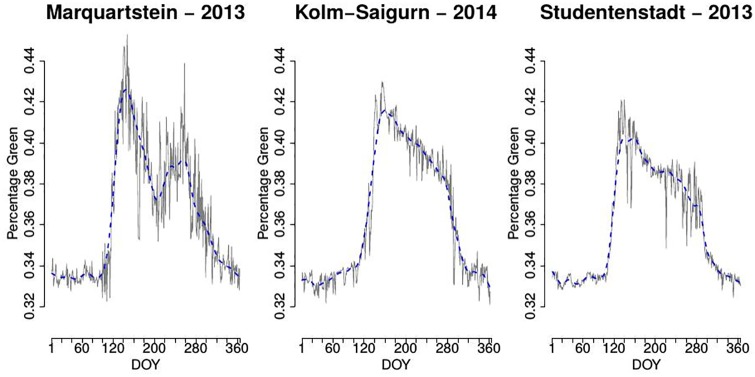
Resulting %greenness time series for sites Marquartstein (left), Kolm-Saigurn (center) and Studentenstadt (right) obtained by the uROI approach.

For the publicly available data from http://www.foto-webcam.eu, where expert-based eROIs were not available, we apply the uROI approach as follows: For each webcam site we compute clusters as described above for different settings, namely for *p* ∈ {12, 24} singular vectors and *k* ∈ {4, 5, 6, 7, 8, 9, 10} clusters for the k-means procedure. This results in 14 partitions of the image and yields in total 98 clusters. For each of the 98 clusters we compare the OC2 of the %greenness time series defined above and thereby identify one optimal cluster for each webcam site. Fig 11 shows the resulting %greenness time series for the optimal cluster of each webcam site. Most interestingly the resulting %greenness time series of the optimal cluster in each picture display different shapes. Whereas in the case of Studentenstadt 2013 and Kolm-Saigurn 2014 a typical deciduous type is achieved, Marquartstein 2013 exhibites after the spring increase an early summer depletion and a second increase from DOY 200 again. We guess that this might be linked to quite dark / underexposed pictures in mid of July 2013. Additionally, careful checking of all pictures reveals a felling of a prominent deciduous tree in the foreground in front of the spruce (cluster 4).

**Fig 12:**
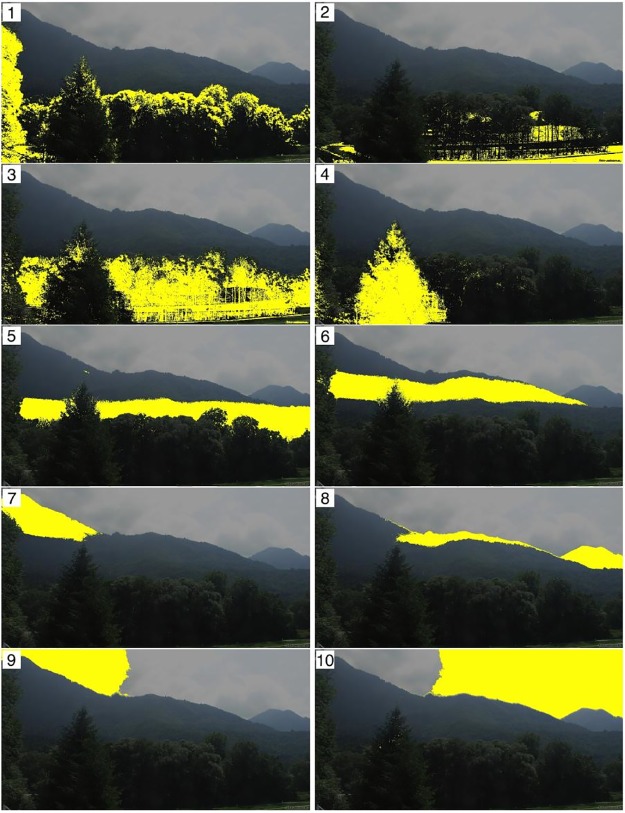
Resulting *k* = 10 clusters from uROI approach for the site Marquartstein, ordered with respect to OC2. Cluster 1 has the highest OC2.

**Fig 13:**
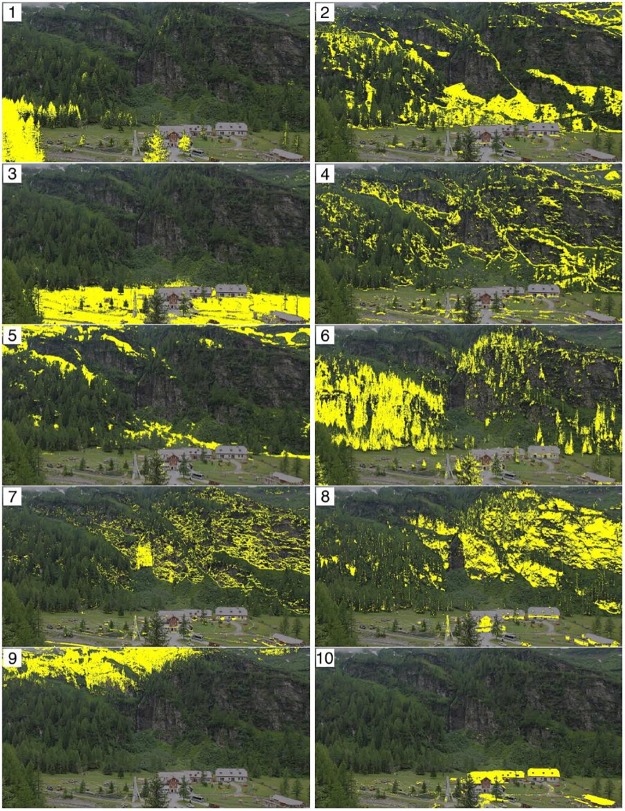
Resulting *k* = 10 clusters from uROI approach for the site Kolm-Saigurn Sonnblickbasis, ordered with respect to OC2. Cluster 1 has the highest OC2.

**Fig 14:**
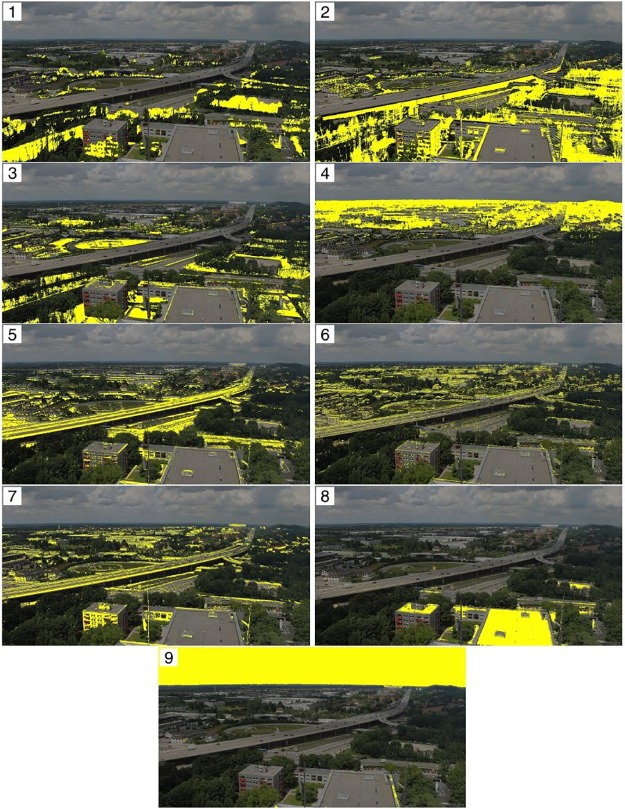
Resulting *k* = 9 clusters from uROI approach for the site Studentenstadt, ordered with respect to OC2. Cluster 1 has the highest OC2.

In Figs 12–14 we show the optimal cluster for each webcam site along with all other clusters from the respective partition by plotting an overlay of a selected original image and the respective cluster in yellow. The clusters are ordered with respect to the information criterion OC2.

Fig 12 shows the clusters for the site Marquartstein. It can be seen that besides two sky clusters (10, 9) four clusters differentiated background vegetation, mainly mixed forests, in elevational belts (8, 7, 6, 5). The foreground vegetation was separated into a single dominating spruce (4) and deciduous vegetation (3), partly also assigned to (2) due to different winter aspects. The optimal cluster (1) consists of the upper crown part of the deciduous forest edge and a tree at the left side of the picture.

Fig 13 shows the clusters for the site Kolm-Saigurn Sonnblickbasis. It displays clusters of meadows in the valley (3), roofs of buildings (10), wall structures and rocks (8) as well as water fall / rocks (7). All vegetation is divided into various clusters from the background (9, likely mugo pine) to deciduous broadleaf vegetation (2, likely alder) in the valley. The optimal cluster (1), however, are deciduous European larch (Larix decidua) turning bright green needles in spring into yellow in autumn.

Fig 14 shows the clusters for the site Studentenstadt-Nord, which is a mixed urban landscape in the northern area of Munich. The clusters comprise sky (9) as well as infrastructure components such as flat roofs / parking space (8), facade and contour lines (7, 6) and streets (5). Clusters comprising predominantly vegetation are background vegetation (4), grass (3), deciduous vegetation including facades and seasonal shadows from the autobahn (2) as well as the optimal cluster (1) with pure deciduous vegetation components.

### Open-access webcams - AMOS

Finally we challenge our methods and apply the uROI approach to a large database of webcams from AMOS. We automatically analyze all webcams which offer images for the year 2015. For most of these webcams, between one and four images per hour are available. We reduce the computational complexity by selecting images of a fixed hour during day time. This is challenging because the timestamp of the images reflects GMT (Greenwich Mean Time) instead of the local time. We restrict the analyses to images with GMT between 5pm and 6pm. This should deliver useful images for webcams located in the United States as well as for webcams located in Europe. We apply the uROI method to all thereby selected webcams. As number of left singular vectors we choose *p* = 12 and as candidates for the number of clusters we define the grid *K* = {4, 5, 6, 7, 8, 9, 10}. Finally, we use OC2 to find the best of the resulting clusters.

To automatically perform the analyses, we proceed as follows. The website offers a python script at http://amos.cse.wustl.edu/dataset which allows to download the data for a given webcam, year and month. We embed this python script in an R script and are thereby able to start the download directly with R. The format of the images is yyyymmdd_hhmmss.jpg. We restrict the analyses to images where the timestamp starts with 17 for the given hour. We distribute the computations on 16 cores on a server with four Intel Xeon E5-4620 CPUs with 2.20GHz and 8 cores each and 528 GB RAM in total. This results in a computation time of 23 days, https://figshare.com/s/074257d9cb42e5e8c643 contains an overview of the results.

**Fig 15:**
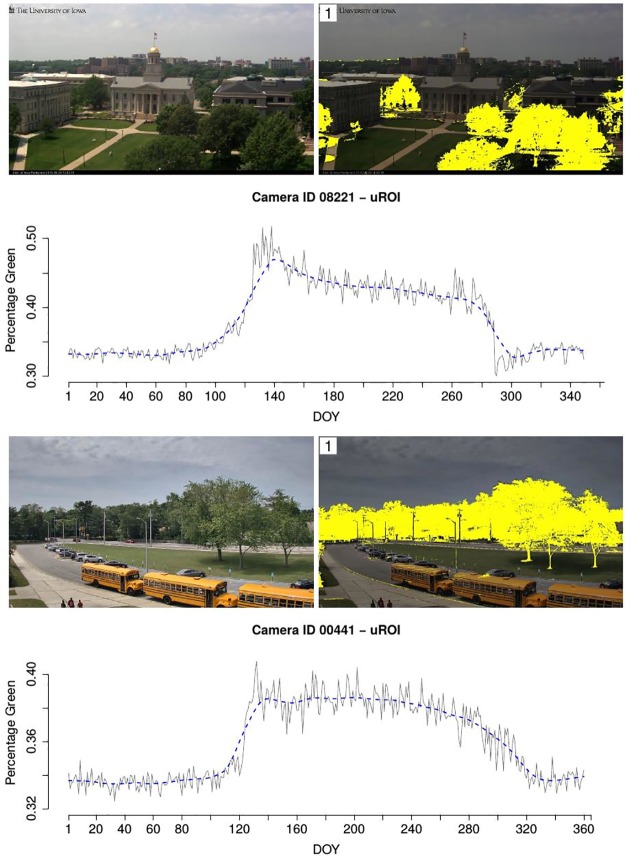
Example image, uROI and %greenness time series in uROI for cameras with ID 8221 (top) and ID 441 (bottom).

**Fig 16:**
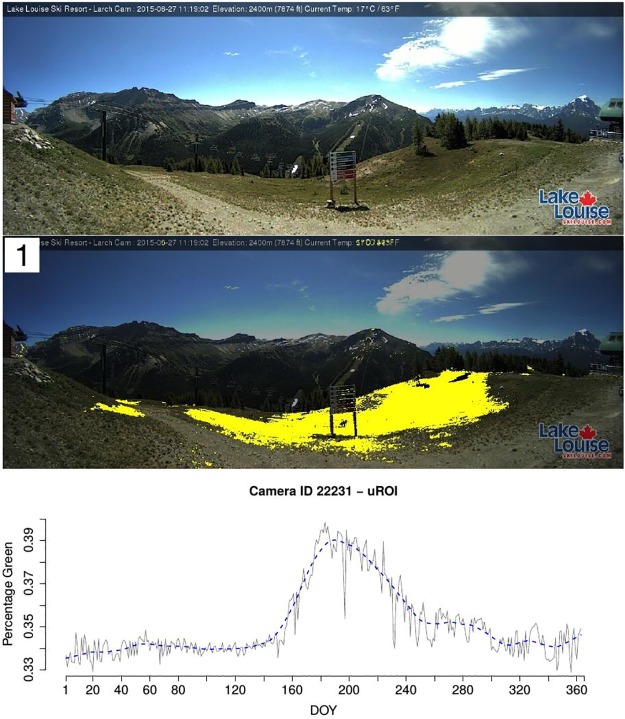
Example image, uROI and %greenness time series in uROI for camera with ID 22231.

In total, for 13988 webcams a uROI analysis was started. For 13095 of these the entire analysis could be carried out without errors, i.e. ROIs and %greenness time series were derived. 9299 webcams offer enough data to compute OC2, for most of the other cameras there are large gaps in the year where no images are recorded. Figs 15 and 16 show example images, final uROIs and corresponding %greenness time series for three selected webcams. In all three cases, the uROI method finds ROIs with high phenological information. For ID 8221 (Fig 15, top) and ID 441 (Fig 15, bottom), the deciduous trees are identified and the resulting %greenness time series show clear structural changes in spring and autumn. For ID 22231 (Fig 16), the grassland in the foreground is defined as uROI since no deciduous trees are captured by the camera. The resulting %greenness time series shows a clear seasonal pattern as well.

## Discussion

**Key findings.** In this paper, we proposed two new methods for the automated processing of webcam images with the purpose of phenological classification.

More precisely, we proposed two alternatives for the expert-based definition of regions of interest (eROIs), namely the semi-supervised regions of interest (sROIs) and the unsupervised regions of interest (uROIs). All approaches lead to ROIs for the image data. Based on these ROIs, %greenness time series can be computed and analyzed. Structural changes in these time series indicate phenological change points. We found that the time series based on the sROI and uROI approaches are as informative as those based on the expert-based eROI approach with respect to two optimality criteria for the example of a scientific webcam in an experimental forest. The different methods were tested to process images from a scientific webcam and to derive the annual course of deciduous vegetation greenness. This led to almost identical results in terms of start, peak and end of season dates. The uROI method was also successfully applied to open-access webcam data from the internet yielding phenologically interesting partitions of the images while clearly separating infrastructure, rocks and buildings. However, the most “deciduous” vegetation type was either the upper crown part (as also shown for the scientifc webcam) or deciduous larch. Quite promising is the fact that different vegetation types (alpine meadow, urban grass) could be clearly separated. Additionally, less (in the case of sROI in the expert version) or no (in the case of sROI in the automated version and in the case of uROI) manual action is necessary to define the ROIs. Hence, we conclude that our new sROI and uROI methods for the definition of ROIs are favorable, especially when working with a large amount of webcams at different stations as shown for the AMOS data with 13988 webcams.

**Strengths and limitations.** One strength of the sROI approach is that ROIs are defined mainly data-driven while the researcher can simultaneously control the definition with setting the pinprick on the favored region of the image. Additionally, the resulting %greenness time series is slightly more informative than with the standard eROI approach.

The first strength of the sROI approach could at the same time considered to be a weakness: Through the fact that the pinprick has to be set manually and at a reasonable position, the approach is not optimal when heading for big data applications and analyses where several hundreds or thousands of webcams shall be analyzed at the same time. However, with the option of setting multiple pinpricks at random locations and selecting the best ROI with the proposed optimality criteria, we also provide a fully automated version of the sROI approach. Nevertheless, this option comes along with an increase in computing time because the analysis has to be carried out for each pinprick. Depending on the given images, a rather large number of random pinpricks can be necessary.

This potential limitation of the sROI approach is not present in the uROI approach: The uROI approach is fully automated and purely data-driven, no manual action is required to define the ROIs and the computing time for this approach is rather small. This property is in particular of great importance for the mentioned big data applications.

Moreover, the resulting %greenness time series are actually as informative as with the eROI approach. This finding has to be emphasized since the definition of expert-based eROIs is accompanied by a large amount of manual work while the sROI and uROI approaches require no manual action at all.

However, there is also a limitation: Though the approaches work promising for the data sets considered here, we cannot blindly guarantee that results are equally satisfying for other data sets. It happens that there are more than only one resulting cluster which is important for the identification of phenological patterns, as shown for example for the data from Marquartstein, see Fig 12, and it could happen that there is no cluster which is pure enough for the identification of phenological patterns. Many scenes show different species and thereby different phenologies. By just selecting the best clusters in the sense of the optimality criterion, this variety is not accounted for in the present approach. It can thereby be advantageous to look at the second best cluster, third best cluster and so on. A further drawback is that an expert would choose very different ROIs sometimes. Even if the presented approach chooses the optimal ROI in the sense of the optimality criterion, this point may be unsatisfying in practice and hence further research will be necessary.

All in all, the results for the 13988 webcams of the large AMOS database are satisfying but nevertheless a blind automatic proceeding is problematic due to the following issues:

- For some webcams, the field of view shifts significantly over the year. This means that some webcams are for example directed towards the north on some days and towards the south on other days. For these cases, meaningful uROIs can obviously not be extracted.
- In other cases, the field of view shifts only slightly. For these cases a registration of the images as preprocessing step would be beneficial. However, as shown in additional results available at https://figshare.com/s/074257d9cb42e5e8c643, the uROI method still finds a meaningful ROI even if the field of view changes over the year.
- As mentioned above, we use images where the timestamp starts with 17 for the given hour. Due to the fact that the timestamp of the images does not refer to the time at the webcam location but to GMT, we get images at night for a variety of locations. Of course, the analysis does not make sense for images at night. Therefore, in a further analysis, the location of the webcams should be extracted from the AMOS website in order to determine the time zone.
- For some webcams, the image quality is rather poor and meaningful uROIs cannot be derived. Of course, the quality of the results depends on the image quality.

We conclude that the presented results for the AMOS data demonstrate that our new methods are suitable for the analysis on a large scale because the computing time is reasonable (23 days for 13988 webcams) and the results are overall satisfying. Even if the above mentioned problems have to be solved in further research, this first analysis seems to be a very promising start.

**Outlook.** This paper is considered as a starting point for a larger project connecting phenologists, computer scientists and statisticians. As next step it is planned to refine the first AMOS analysis and to solve the problems mentioned above. Thereby, season onset dates should be derived for each webcam location and year yielding high-resolution spatio-temporal data. Then, it could be investigated how the season onset dates change over the years, and spatial patterns of these changes could be analyzed. Furthermore, the question should be addressed if these changes are related to the global climate change.

From a user’s perspective it would certainly be of interest to implement the Bayesian multiple change point analysis in the R package phenofun. (The R package phenofun contains all relevant functions to use the presented methods and is available on figshare at https://figshare.com/s/a93e26f68fdcab0ae9b3 (windows binaries) and https://figshare.com/s/22172055702e2cb31114 (source package).) Then, not only %greenness time series inside the final ROI but also season onset dates could be derived automatically and with a single R script. Furthermore, the question of uncertainty of the estimated season onset dates should be addressed.

## Conclusions

We conclude that with the proposed new methods for the automated processing of webcam images, we paved the way for analyzing webcam data on a large scale. Especially with the fully automated and purely data-driven definition of regions of interest, we are now able to analyze a large amount of webcam data from many different stations with low manual effort, as shown for 13988 webcams of the AMOS database. We hope that our work can be a relevant contribution for the application and analysis of webcam images for phenological questions.

## Supporting information

S1 Code Example code for R package phenofun. This.zip file contains executable example code for performing automated sROI and uROI analyses and for reproducing the AMOS analysis.
(ZIP)

S1 Fig Detailed results for webcam ID 1 from AMOS. This file contains the resulting clusters and %greenness time series for webcam ID 1 which shows a significant change in the field of view over the year.
(PDF)
