# Supplementary material for: Automated processing of webcam images for phenological classification
Source: PLoS One. 2017 Feb 24;12(2):e0171918. doi: 10.1371/journal.pone.0171918 (PMC5325214; doi:10.1371/journal.pone.0171918)
Supplement: S1 Fig — This file contains the resulting clusters and %greenness time series for webcam ID 1 which shows a significant change in the field of view over the year. (PDF) [file pone.0171918.s002.pdf]

uROI analyses of AMOS data - ID 1

Ludwig Bothmann

July 21, 2016

Figure 1 shows the uROI (cluster 1, top left) for webcam ID 1 of the AMOS data together with all other clusters from the best partition. Clusters are ordered with respect to optimality criterion OC2, cluster 1 has the highest OC2. Figure 2 shows the corresponding %greenness time series for each cluster, also ordered with respect to optimality criterion OC2.

Example images can be found at `http://amos.cse.wustl.edu/camera?id=1#20150916_203026` and `http://amos.cse.wustl.edu/camera?id=1#20150917_203042` showing that the field of view changes over the year.

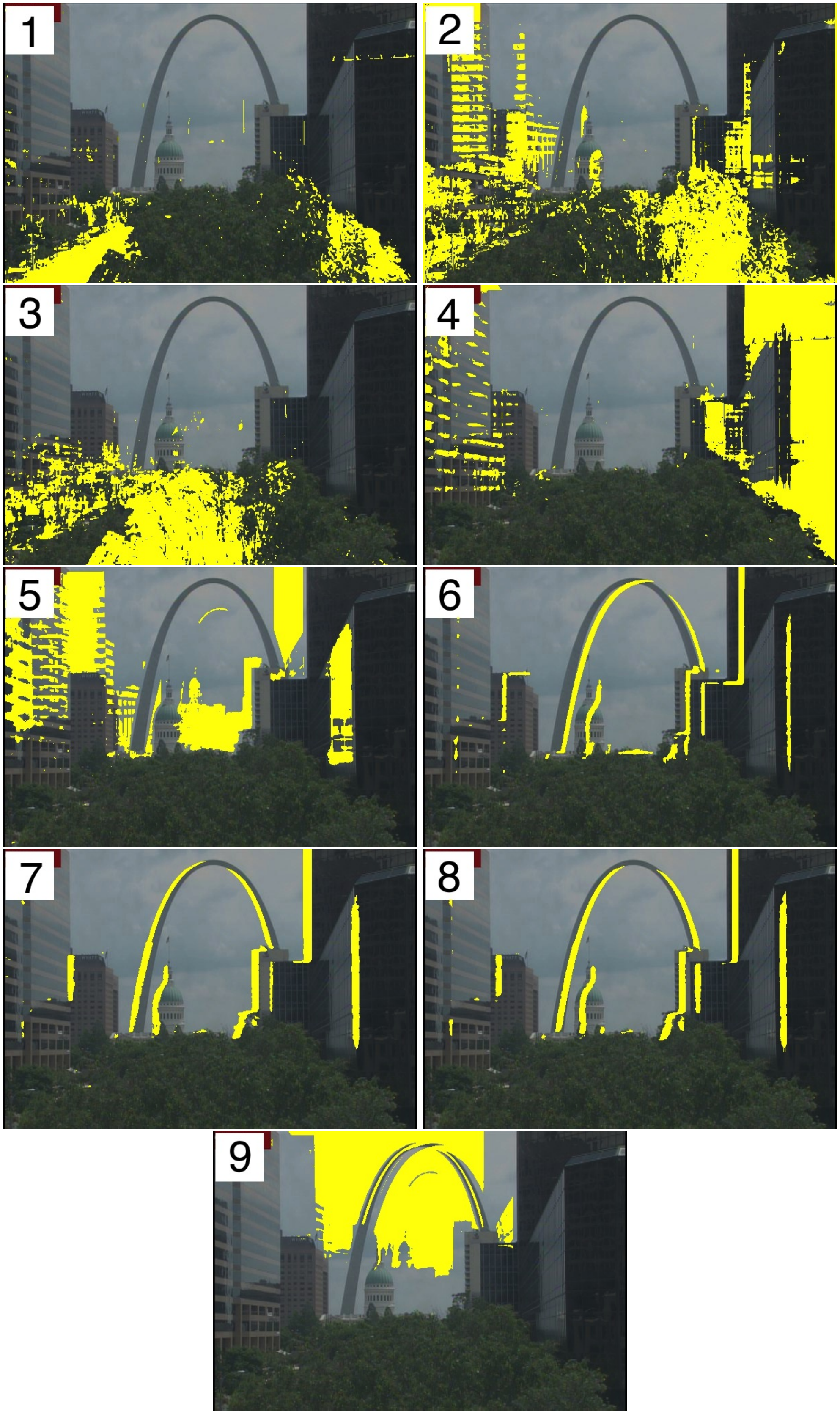

Figure 1: Resulting $k = 9$ clusters from uROI approach for ID 1 of AMOS, ordered with respect to OC2. Cluster 1 has the highest OC2.

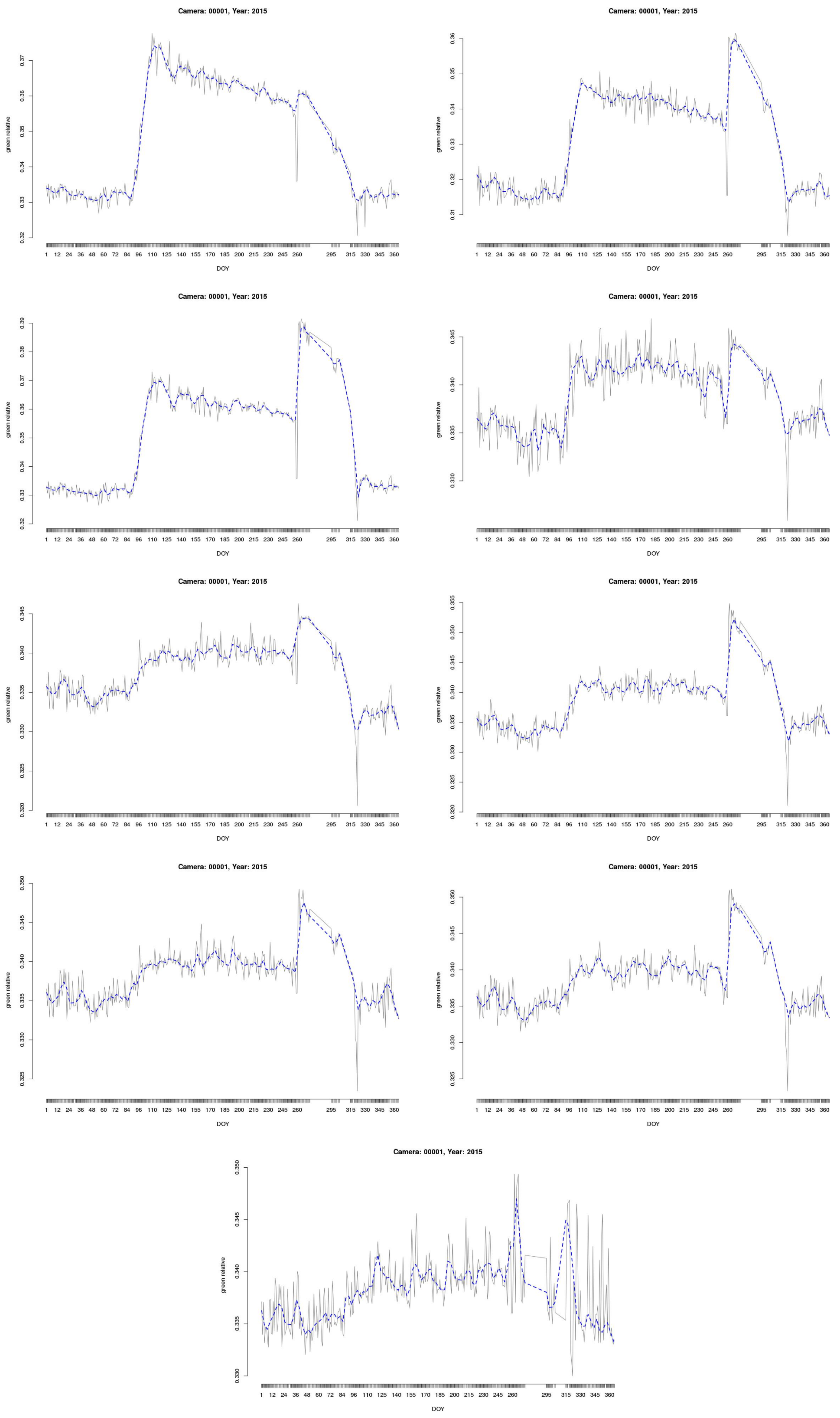

Figure 2: %greenness time series for $k = 9$ clusters from uROI approach for ID 1 of AMOS, ordered with respect to OC2. Top left time series has the highest OC2.
